# Identification of Maya ruins covered by jungle using Sentinel-1

**DOI:** 10.1038/s41598-024-53068-2

**Published:** 2024-02-08

**Authors:** Florent Michenot, Israel Hinostroza, Regis Guinvarc’h, Laetitia Thirion-Lefevre

**Affiliations:** SONDRA, CentraleSupélec, Université Paris-Saclay, 91190 Gif-sur-Yvette, France

**Keywords:** Imaging techniques, Archaeology

## Abstract

Archaeologists commonly use airborne LIDAR technology to produce 3D models of structures, even when obscured by a forest canopy. However, this technology has a high cost, both from the plane itself and from the processing of the LIDAR point cloud. Furthermore, this technique can only be used over limited regions. This paper proposes a technique that uses SAR satellite imagery to identify man-made structures hidden by a forest canopy. To do so, we exploit the Ascending and Descending passes of Sentinel-1 so that we obtain two images of the candidate site but from different sight directions. Because of cardinal effects, a large enough building will sign differently from the comparatively isotropic forest canopy it is obscured by. Practically, the technique is based on the ratio of backscattered intensity from these two illumination angles and is well adapted for large areas. The advantages and shortcomings are discussed for the specific case of Sentinel-1 SAR images over two Maya archaeological sites in Central America. Our analysis shows that SAR satellite imagery might provide a free, global-scale way of preselecting sites with large or tall structures to complement LIDAR technology.

## Introduction

In addition to fieldwork, archaeologists today use different technologies to discover the remains of man-made objects, such as Airborne *LIght Detection And Ranging* (LIDAR, also known as Airborne Laser Scanning or ALS). Its performance, both in terms of resolution and forest penetration^1^, has made it an alternative to earlier techniques for more than 15 years. In particular, recent technological progress has led to a further refinement in image resolution, which in turn has led to the relatively rapid spread of this technique, especially among archaeologists^2–4^. However, the prohibitively high cost of airborne LIDAR missions restricts its use to relatively small areas and to known or suspected sites.

Synthetic aperture radar (SAR) images can potentially be a low-cost global-scale alternative to LIDAR if provided by a spaceborne sensor. SAR has already been used for detecting man-made objects^5^, and in particular buildings^6^ and roads^7^, for at least 30 years^8^. The techniques used for building detection are diverse: statistical approaches^9^, polarimetry^10^ or polarimetric interferometry^11^, and machine learning algorithms^12,13^. When buildings are obscured by forest, SAR penetration properties can be exploited. Some authors proposed visual photo-interpretation for building detection^14,15^.

Additionally, SAR is a useful tool available to archaeologists even for other applications than building detection. Through interferometry (InSAR), it is possible to create Digital Elevation Models (DEM) for large areas. One notable example is SRTM^16^, a free global-scale DEM provided by NASA at a 30m resolution. This overview of a region’s topography enables archaeologists to find plausible sites for human settlements or explain why sites were abandoned and populations migrated^17^. InSAR also enables the monitoring of ground movements and subsidence affecting cultural heritage sites^18^. Polarimetry (PolSAR) is another aspect of SAR that archaeologists benefit from. Either directly^19^ or through the combination of SAR sensors working at different frequencies^20^, it is possible to reveal buried structures in deserts or other dry open areas.

SAR benefits from exploiting buildings’ anisotropy, which leads to the non-uniformity of their radar backscatter when measured from different azimuthal angles^21^. As the SAR synthesis process returns a unique azimuth value for each row of pixels, these multiple azimuthal points of view are generally obtained using sub-apertures on either polarimetric^22,23^ or non-polarimetric^24^ data. However, using sub-apertures degrades the image’s azimuthal resolution, and the angular range is limited by the antenna’s real aperture. As this aperture is quite small for spaceborne SAR ($0.23^\circ$ for Sentinel-1), the benefits of using sub-apertures are pretty limited in our case.

Anisotropy causes shadows. This is still true for SAR shadows, whose shape depends on the angle of incidence and the azimuthal angle. By detecting the shadow, one can leverage how the building breaks the isotropy of the scene (along the range axis) instead of the anisotropy of the structure itself^25^. This detection can reveal a building even when obscured by a jungle^15^.

Forest penetration (FoPen) by a radar signal is easier at lower frequency (typically P- and L-band^26^). At higher frequencies (C-band in our case), SAR is generally considered inadequate^27^. Some hypotheses must then be made: either there is no penetration, and any building signature detected is due to a reproduction by the forest canopy of the shape of the structure below, or there is some penetration, in which case backscattered signal from the ruin (surface scattering or possibly double-bounces) is received by the SAR sensor. In either case, the presence of the building leads to an increase in the anisotropy of the scene.

This paper processes multiple images taken by the same sensor from different points of view (multiple flights of a plane, multiple orbits of a satellite), thereby combining range and azimuth anisotropy to identify possible buildings, even through forest canopies. With this method, archaeologists could easily preselect candidate sites worldwide and for free before further exploration campaigns.

For testing the method, we studied some warehouses located in Saint-Martin-de-Crau, France, and the Pyramid of the Sun in Teotihuacan, Mexico. We validated the technique on two areas of interest: the archaeological site of Lamanai in Belize and the Calakmul Biosphere Reserve in Mexico. Both are in Central America, as the region contains dense jungle, rugged terrain, and hidden Maya structures.

## Study areas and datasets

### Study areas

The main archaeological site studied in this paper is Lamanai, in northern Belize. It is a known Maya site, open to tourists, with its own local museum. Parts of the site have been cleared of trees, and the buildings there were cleaned but not restored. This was the case for High Temple (Fig. 13). Other parts of the site were mapped but left to the jungle. This area was the most interesting, as it provided us with known and more or less geolocated buildings hidden by the forest canopy. In particular, structures P8-1 and P9-25 (Fig. 14) were large enough to be clearly visible using the technique presented in this article while still being undetectable from satellite optical imagery.

We also used our technique on suspected sites identified by LaRocque^15^ in the Calakmul Biosphere Reserve, at Mexico’s border with northern Guatemala. They are called Site A, B and C (Fig. 17). The area also contains several known Maya sites. Although we focused our work on Central America, our approach is generic: it only requires access to SAR time series taken from two different directions over your area of interest.

**Figure 1:**
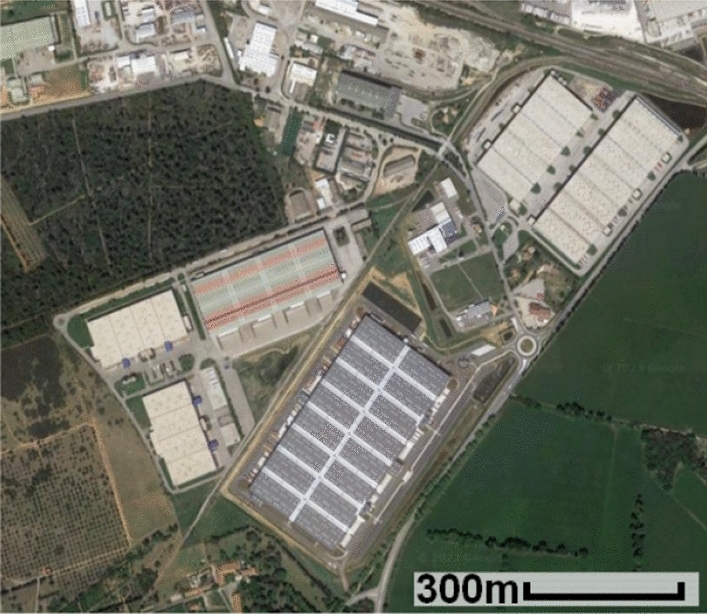
Optical image^28^ of the warehouses used to illustrate the technique. The footprint of the picture is identical to the one in the radar images. North is up.

We have also looked at two areas without trees: some warehouses (Fig. 1) in Saint-Martin-de-Crau, southern France, and the Pyramid of the Sun in Teotihuacan (Fig. 11), Mexico. The warehouses were useful because of their simple geometry, large size and the probable presence of double bounces. The pyramid was our next step on our way toward hidden pyramids, as its geometry was expected to be closer to theirs than the warehouses’, even though it was not built by Mayans.

Information about the AoIs was summarized in Table 1.

**Table 1:** Summary of the areas of interest studied in this article.

Area Name	Coordinates	Area Type
Lamanai	17.770, − 88.650	Site covered by jungle
Site A	17.990, − 90.170	Site covered by jungle
Site B	17.895, − 90.050	Site covered by jungle
Site C	17.920, − 90.140	Site covered by jungle
Warehouses	43.620, +04.790	Group of warehouses near fields
Pyramid of the Sun	19.693, − 98.845	Lone pyramid in flat terrain

To our knowledge, meso-american buildings are not perfectly aligned with the cardinal directions. This is for instance the case of the structures shown in this article.

### Sentinel-1

We looked for a SAR satellite capable of providing us with SAR time series from different points of view over our areas of interest. Some candidates are presented in Table 2. For forest penetration (FoPen) applications, a lower frequency is preferred (P- or L-band^26^). For building detection purposes, HH is preferred (because of its increased backscatter intensity in the presence of double-bounces^29^, for example). Among the satellites that provide free data, only Sentinel-1 has time series from different directions (called *ascending* and *descending*, due to the direction the satellite is moving with regard to the ground). It operates at a higher frequency than we would have liked, and generally does not generate HH images over land.

**Table 2:** Summary of recent SAR satellites capabilities, adapted from^18^. When multiple beam modes were provided, only “Strip Map” was kept (or “Interferometric Wide” for Sentinel-1). time series availability depends on the existence of a long enough (15+ images) dataset over Guatemala from both the ascending and descending directions. The age of the data is irrelevant for our application. Indicated polarization might not be available for all beam modes.

Name	Band	Polarization	Resolution (m)	time series availability	Free
Radarsat-1	C	HH	10-100	No	Yes
PALSAR	L	Full	10-100	No	Yes
Radarsat-2	C	Full	3-100	Yes	No
COSMO-SkyMed	X	Single or Dual	3	Yes	No
TerraSAR-X	X	Single or Dual	3	Yes	No
Sentinel-1	C	Single or Dual	5x20	Yes	Yes
PALSAR-2	L	Full	3/6/9	Yes	No

**Figure 2:**
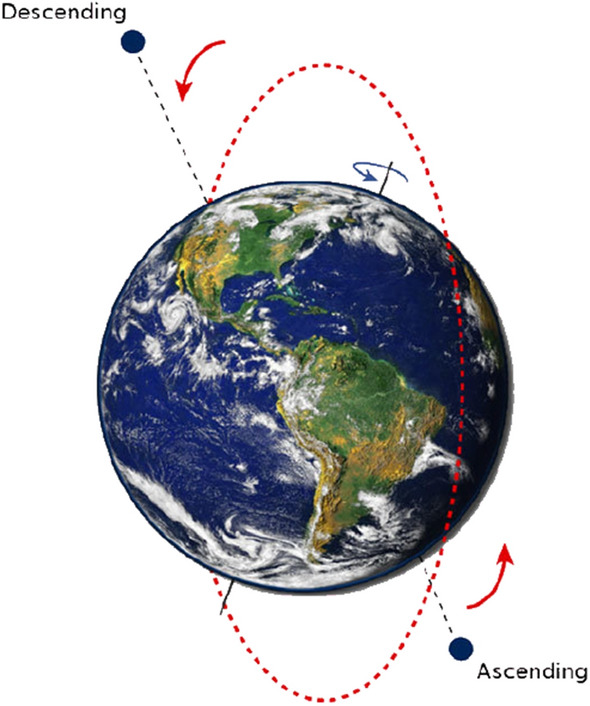
Illustration of Sentinel-1 orbit © SkyGeo. A *descending* path follows an *ascending* path, and vice-versa.

In this article, Sentinel-1 refers to a pair of C-band SAR imaging satellites. Sentinel-1A was launched in April 2014, and Sentinel-1B (recently rendered inoperative) in April 2016.

These satellites from the European Space Agency (*ESA*) have a heliosynchronous orbit with a 12-day repeat cycle, meaning that a point on the planet is in range of a particular satellite every 12 days but visible by either every 6 days. This makes Sentinel-1 one of the first SAR sensors to give access to dense time series over the entire globe for free.

Moreover, each satellite actually covers the same spot twice during each orbital repeat cycle: once when going from the South pole to the North pole (*ascending* path), and once when going the other way around (*descending* path) 6 days after. Because they are right-looking sensors, this gives us two distinct points of view over a target, fulfilling the requirement for exploiting anisotropy. The line of sight comes roughly from the West on ascending images and the East on descending images.

### Dataset characteristics and Preprocessing

In this study, we make use of Sentinel-1 *Ground Range Detected* (GRD) images taken with the *Interferometric Wide* (IW) swath mode. The images have a square resolution of about 20 m and a square pixel spacing of 10 m.

This square pixel spacing is due to the multi-looking used by ESA during the GRD image generation. The multi-looking factor they used was 5 in range and 1 in azimuth.

The polarization available in this mode over most places is VV+VH. We choose to use VV-only. The technique can be indifferently applied to VH.

**Figure 3:**
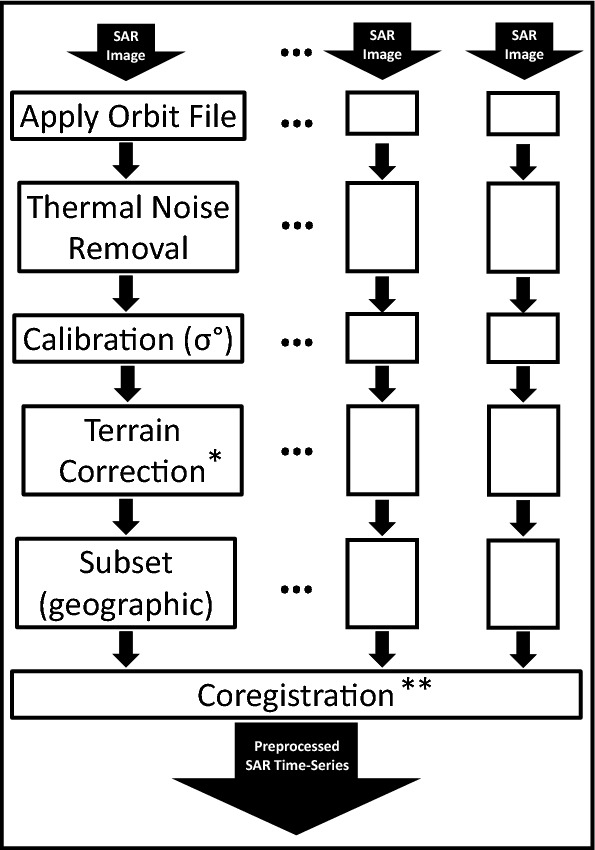
Flowchart describing the preprocessing protocol used. The protocol comes from a tutorial^30^ by the European Space Agency. It must be applied to each direction separately.

Preprocessing steps are required for time series analysis, particularly coregistration and georeferencing. In this paper, they have been done using ESA’s SNAP tools. The preprocessing operation themselves were taken from an ESA tutorial^30^. A flowchart of the preprocessing is shown in Fig. 3. Note that the time series from the two directions must be preprocessed separately. More details and justifications can be found in the tutorial. We do have some recommendations:

* In the Terrain Correction step, we recommend disabling “Radiometric Normalization” to preserve the original intensities. For the same reason, “Nearest Neighbour” image resampling method should be used.
* Still during Terrain Correction, we recommend using the UTM coordinate reference system instead of the default one (in the “Map Projection” menu), because the latter will use the angular pixel spacing (in ^∘^) leading to potentially not-square pixels whereas the former uses the linear pixel spacing (in m).
** The ESA tutorial simply “stacks” the different images. This is generally enough for GRD images. The more computation-intensive “coregistration” algorithm can also be used. Note that, for GRD images or for intensity-only applications, “coarse” coregistration is enough.

** If you do use coregistration, be aware that it can fail on areas of dense forest. In our case, we have used larger subsets also covering non-forest zones to enable the coregistration.
** We recommend using the “Nearest Neighbour” resampling method whenever interpolation is needed during the coregistration or the stacking.
** Be sure to choose a master image of the same size in both directions, otherwise you will not be able to compute the ratio.

The time series we are using are composed of every image taken between 18/11/2019 and 18/11/2020 (one year) over each target area but are separated by orbits (and directions) to keep the different incidence angles apart. The stacks generally include around 60 images each (when both Sentinel-1 A and B are available). This can drop to about 30 images depending on the area surveyed (when only one of the two satellites’ output is provided by ESA).

This 30-date criterion impacts the speckle filtering performance and reduces the effects of the variation in the scene moisture along the year.

After the preprocessing is done, the pixels represent $\sigma ^\circ$ and are georeferenced. Because of this, it is possible to overlay the time series taken from the ascending and descending directions together. A particular pixel represents the backscattering from the same area on both images, except for some geometrical artifacts. We propose to leverage that spatial diversity to detect man-made structures.

## Method

In this section, we present a generic remote sensing building identification method before detailing the particularities of its SAR implementation.

### General principle

**Figure 4:**
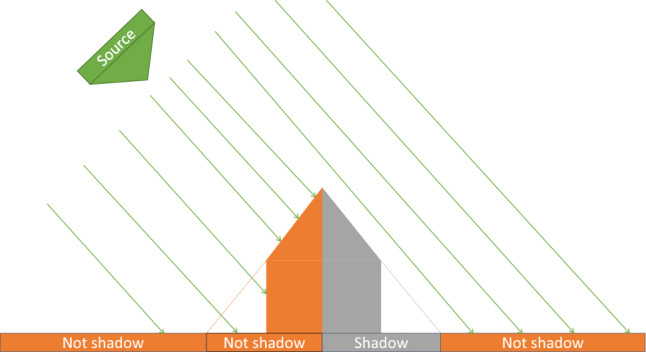
Illustration of the illumination of a man-made structure from the left.

**Figure 5:**
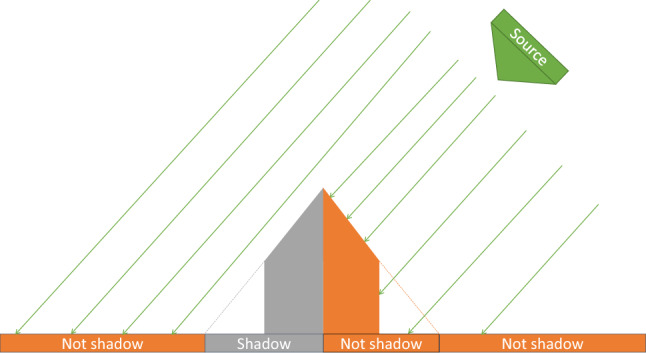
Illustration of the illumination of a man-made structure from the right.

**Figure 6:**
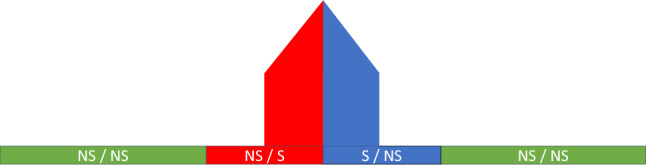
Illustration of a *ratio image* between both directions. The left and right parts of the building can be distinguished both from each other and from the neighboring environment.

For simplicity, let us first consider a building in flat terrain. When illuminated from a particular direction (different from nadir), the structure will cast a shadow (Fig. 4). Any point in the scene is then either a shadow (S) or not a shadow (NS). If the illumination source moves around the object, so does the shadow on the opposite side of it (Fig. 5). Suppose a sensor sensitive to this shadowing effect was to capture the scene at two different times, with the illumination source being at two different positions. It should then be possible to generate a *ratio image* (Fig. 6) highlighting the difference caused by the shift in illumination direction.

For convenience, let us focus on the case where the two illumination directions are on opposite sides of the building. The problem becomes planar: the directions can now be referred to as *left* and *right* from the observer’s point of view. Let us divide the scene into three distinct areas: the *left* part of the building, the *right* part, and the neighboring environment.

When illuminated from the left, the right part of the building is shadowed. The left part and the environment are not. Conversely, when illuminated from the right, the left part of the building is shadowed, but the right part and the environment are not. By computing the ratio, pixel-by-pixel, between an image where the scene is illuminated from the left and another image where it is illuminated from the right, a single *ratio image* is obtained. This image is made up of the following values:

- The left part of the building contains *NS*/*S*.
- The right part of the building contains *S*/*NS*.
- The environment contains *NS*/*NS*.

If we suppose that the quantity returned by the sensor for *NS* is greater than for *S*, then these three ratios are respectively greater than 1, smaller than 1, and equal to 1. On a logarithmic scale, this would correspond to, respectively, a positive value, a negative value, and zero.

In principle, this method should be usable with any imaging system, as long as it is sensitive to the shadows cast by buildings and that different illumination directions are available. We will now apply it using SAR.

### Ascending/descending ratio

We will start with SAR images of a group of large warehouses surrounded by fields, a small forest and the edge of a town (Fig. 1). A SAR sensor is an **active** sensor: it is its own illumination source. The walls and slopes of the structure facing the sensor should strongly reflect the radar signal by single, double, or even multiple bounces (high-intensity backscatter, HI). Parts of the building and terrain on the other side will be shadowed from the satellite’s point of view and will, therefore, have a low backscattering coefficient (S). As a reminder, in the case of Sentinel-1, it is the western and eastern faces which will be either strongly illuminated or shadowed. The terrain around the building will also produce a low intensity, depending primarily on its roughness (low-intensity backscatter, LI). It should, however, still be higher than the shadows. It is safe to assume the following:

1 $$\begin{aligned} HI> LI > S \end{aligned}$$

**Figure 7:**
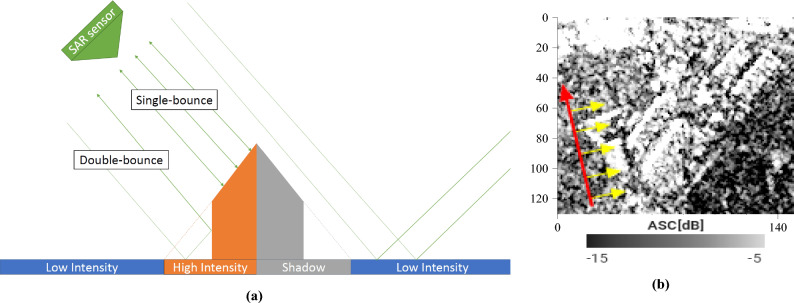
Example of an ascending (illuminated from the West) SAR image: (**a**) the scattering mechanisms in play over a man-made structure, from the ascending direction (**b**) an ascending SAR image of some warehouses, with speckle. Image taken by Sentinel-1, preprocessed using SNAP^31^, processed using Python. The axes for the image are in pixels. One pixel is 10 m by 10 m. North is up. The satellite trajectory was indicated in red, some SAR lines of sight were drawn in yellow. Note the brightness of the western sides and the shadows, particularly visible between the two smaller warehouses in the top right corner.

The scattering mechanisms are shown in Fig. 7a, and the corresponding SAR image of the warehouses is displayed in Fig. 7b.

**Figure 8:**
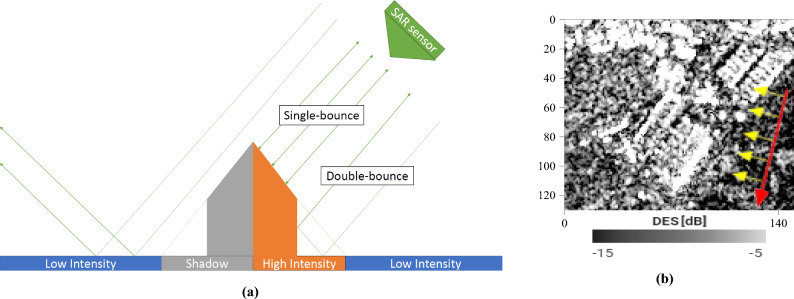
Example of a descending (illuminated from the East) SAR image: (**a**) the scattering mechanisms in play over a man-made structure, from the descending direction (**b**) a descending SAR image of some warehouses, with speckle. Image taken by Sentinel-1, preprocessed using SNAP^31^, processed using Python. The axes for the image are in pixels. One pixel is 10 m by 10 m. North is up. The satellite trajectory was indicated in red, some SAR lines of sight were drawn in yellow. Note the brightness of the eastern sides and the shadows, particularly visible between the two smaller warehouses in the top right corner.

Were we to look at the building from the opposite direction, the scattering mechanisms **for each part of the building** would now be different. More importantly, the backscattered intensities (part by part) would be different from the previous direction (Fig. 8).

**Figure 9:**
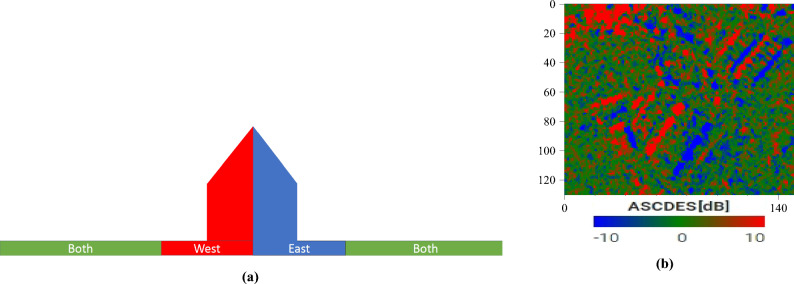
Illustration of the technique: (**a**) the different colored zones, corresponding to the western (red) and eastern (blue) sides of the building, and to both its center and the neighboring ground (green) (**b**) Example of the processing of the SAR images of the warehouses. Image taken by Sentinel-1, preprocessed using SNAP^31^, processed using Python. The axes for the image are in pixels. One pixel is 10 m by 10 m. North is up. Note the speckle that creates red and blue areas over the ground (that should be uniformly green).

In the case of Sentinel-1, the two directions are called *ascending* and *descending* (Fig. 2). The *ascending* path corresponds to the parts of the orbit where the satellite goes from South to North, and the *descending* path to where it goes from North to South. The difference $\Delta \sigma ^\circ$ (in dB) between an ascending and a descending image (see Equation 2) is strong for pixels where the scattering mechanisms lead to a great variation between the two directions. Conversely, a near-zero difference implies that the mechanisms are identical from both directions, i.e. that the scene is locally symmetrical with regard to these directions. The difference image, therefore, highlights pixels where the East-West symmetry is broken (Fig. 9). As earlier, the difference image can be divided into three distinct areas:

- The western part of the building contains $HI_{dB} - S_{dB}$.
- The eastern part of the building contains $S_{dB} - HI_{dB}$.
- The environment contains $LI_{dB} - LI_{dB}$.

Because of Eq. 1, these three differences are, respectively, positive, negative, and zero.

2 $$\begin{aligned} \Delta \sigma ^\circ (x, y, t_1, t_2) = 10\log _{10}\left( \frac{\sigma ^\circ _a(x, y, t_1)}{\sigma ^\circ _d(x, y, t_2)}\right) = \sigma ^\circ _{a_{dB}}(x, y, t_1) - \sigma ^\circ _{d_{dB}}(x, y, t_2) \end{aligned}$$

with $\sigma ^\circ _a(x, y, t_1)$ and $\sigma ^\circ _d(x, y, t_2)$ the backscattering coefficients for the (*x*, *y*) pixel as seen from (respectively) the ascending and descending directions, at respectively the dates $t_1$ and $t_2$, and $\Delta \sigma ^\circ (x, y, t_1, t_2)$ the difference (in dB) between said coefficients.

Because of the three areas we expect to find (West, Both and East in Fig. 9a), we recommend displaying the difference images using a three-colored linear colormap. In this article, we have used red, green and blue. This is NOT an RGB composite. Some assumptions were made when defining the colormap: it is centered on 0 dB (on average, similar intensity from both directions) and is symmetrical (neither direction is preferred, on average over the whole image). In the article, the bounds are arbitrarily always set to $\pm 10$ dB, as we have found it to be visually appealing over our various study areas. The reader is free to change this value, either arbitrarily or by using statistics.

For the sake of simplicity, the SAR geometry was simplified in this section. Accounting for layover, foreshortening and the true shape of SAR shadows makes comprehension harder without bringing significant improvements to the faithfulness of the model (at least when working with Sentinel-1). However, for the sake of rigour and for future high-resolution works, we have included in the Supplementary Material more detailed figures meant to replace figures 7 through 9.

In this section, we assumed that the only difference between the two images comes from the shift in illumination direction, as we are considering tropical forests with a significant moisture all over the year. However, SAR images are plagued by speckle noise. The proposed technique will preserve this noise, which could make the $\Delta \sigma ^\circ$ image challenging to interpret. This should be corrected.

### Temporal speckle filtering

One possible way to reduce speckle is *multilooking*^32^, i.e., dividing the entire image into several smaller overlapping *looks* to obtain multiple points of view and (statistical) observations of a target. By then averaging the looks, you get an image with reduced speckle at the cost of spatial resolution.

**Figure 10:**
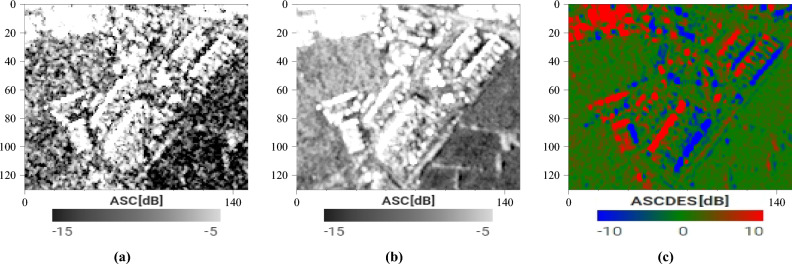
Illustration of the impact of speckle filtering: (**a**) the ascending SAR image from Figure 7b (**b**) temporal mean of the ascending SAR time series of the warehouses (**c**) Ascending over Descending ratio of the temporal averages. Note the uniformly green area for the flat terrain. Images taken by Sentinel-1, preprocessed using SNAP^31^, processed using Python. The axes for the images are in pixels. One pixel is 10 m by 10 m. North is up.

Instead of using subsets of the same image, we advocate using different full-resolution images taken at different times (see Equation 3). This way, we can get the same speckle reduction without decreasing spatial resolution. We can easily achieve this with Sentinel-1 data, as shown in Fig. 10. Note that the GRD images we use are already multi-looked, and have therefore a slightly reduced speckle to begin with.

3 $$\begin{aligned} \sigma ^\circ _D(x, y) = \frac{1}{N_D}\sum ^{N_D}_{i=1}\sigma ^\circ _D(x, y, t_i) \end{aligned}$$

with $N_D$ the number of images from the *D* direction used, $\sigma ^\circ _D(x, y, t_i)$ the backscattering coefficient for the (*x*, *y*) pixel on the $i^{th}$ image from the *D* direction, and $\sigma ^\circ _D(x, y)$ the averaged value.

However, temporal information is lost. Targets only present during a few dates cannot be detected, as their backscattering gets averaged out. In the context of our application, this is not a problem: the heritage sites won’t move across the time series. In fact, this is actually beneficial, as it will improve the contrast between pixels exhibiting consistently high backscattering across the stack and those showing significant variability.

### Canonical case: pyramids

We have decided to use our technique to find archaeological sites hidden in the Central American jungles. We were expecting to see some pyramids there and have therefore looked for known pyramids in open terrain to identify the specifics of their signatures. It is a good first step, although the impact of the canopy will naturally not be taken into account here.

**Figure 11:**
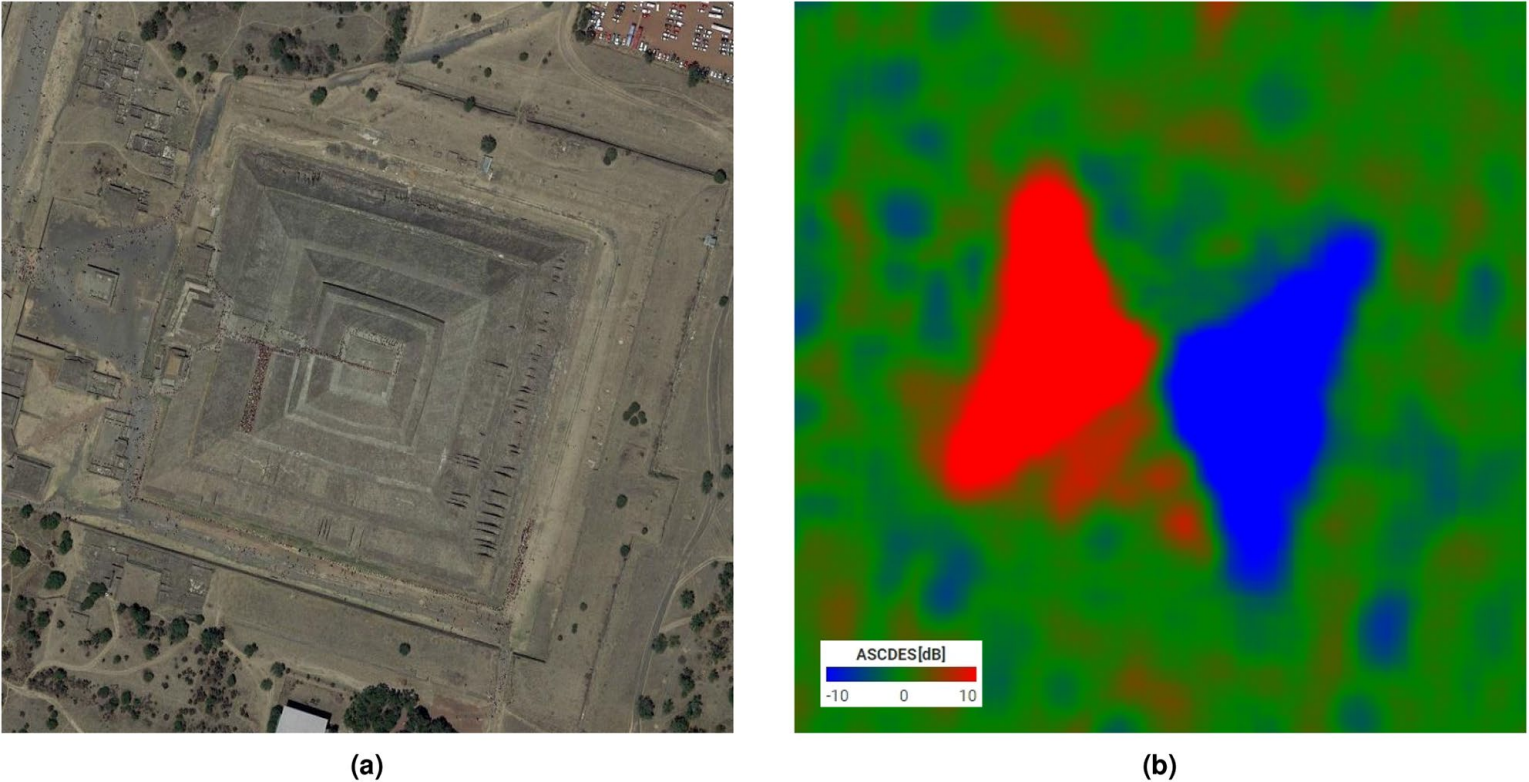
Illustration on a pyramid in open terrain: (**a**) Optical image of the pyramid of the Sun^28^ (**b**) Ascending over Descending ratio over the same area. Image taken by Sentinel-1, preprocessed using SNAP^31^, processed using Python. North is up.

The pyramid of the Sun (Fig. 11) is situated in Teotihuacan, Mexico. It is 200m wide by 70m tall and is slightly turned compared to the cardinal directions.

The western and eastern sides are perfectly delimited. The southern side is colored red, though not perfectly, and the northern side is somewhat more blue than green. The misalignment of the pyramid actually makes our technique more effective as there are no fully symmetrical sides, i.e., they are all at least hinted at on the RGB picture.

### Detection through the forest canopy

Sentinel-1 works in C-band, a frequency band generally considered unsuitable for Forest Penetration (FoPen) applications, as lower frequencies are preferred due to their higher penetration capability^26,33^. Detection of hidden structures should then be difficult unless the forest canopy is locally sparser or there exist holes in the canopy^34^.

**Figure 12:**
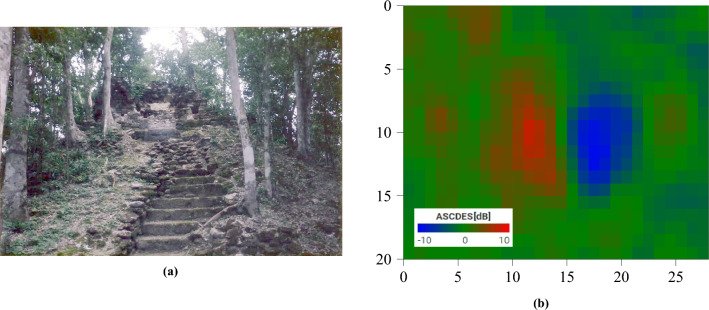
(**a**) Picture of a pyramid from the archaeological site of Nakbe, Guatemala^35^. Trees can be seen growing on the structure. (**b**) AscDes signature of a pyramid in Nakbe covered by trees. Image taken by Sentinel-1, preprocessed using SNAP^31^, processed using Python. The axes for the image are in pixels. One pixel is 10 m by 10 m. North is up.

The method relies on an intensity difference between the ascending and descending paths. If no such difference exists, then detection isn’t possible. A flat, impenetrable forest canopy would appear predominantly green. And yet, in some cases, we see an intensity difference. We propose two hypotheses:

- No penetration happens. However, trees grow on the structures as seen in Fig. 12a. If the trees are of homogeneous height, then the canopy reproduces the shape of the buildings. Surface scattering by the canopy emulates the one from the ruins it covers. Evidence of this happening for large buildings is shown in Figure 12b. It is doubtful that this phenomenon also occurs in small ruins.
- Some penetration does happen. The received signal contains the fields backscattered by the building (direct surface scattering and ground-building interactions), along with a contribution from the canopy.

We believe both hypotheses can be true simultaneously: trees growing on structures would always benefit the technique, as long as we do not have complete canopy penetration (in which case it would have no effect), and partial penetration would explain the uncovering of small structures.

## Results

We have applied our technique to a known Maya archaeological site hidden in the jungles of northern Belize: Lamanai.

**Figure 13:**
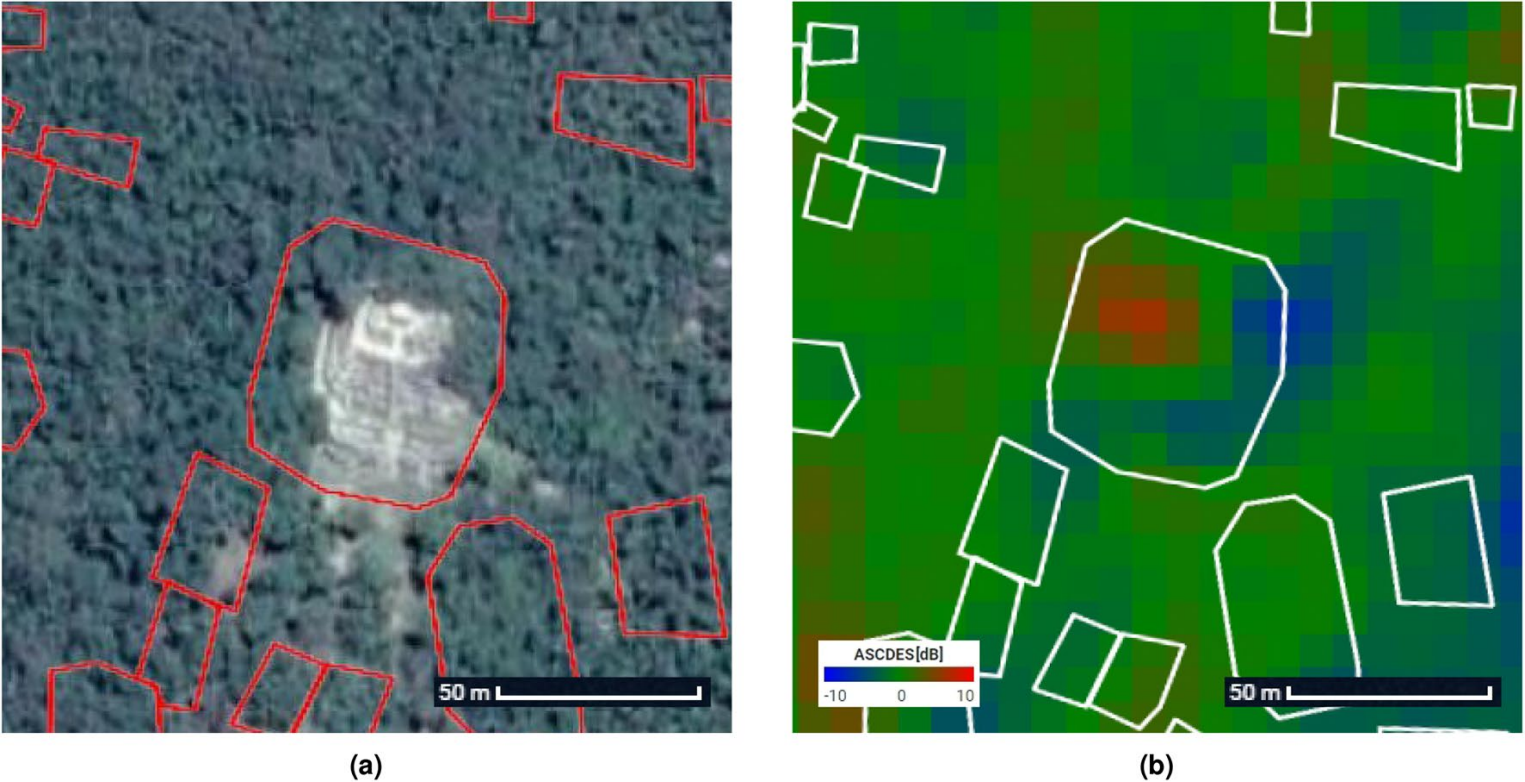
High Temple (aka Structure N10-43): (**a**) optical image^28^ with overlaid map of the structures (red) (**b**) AscDes ratio with overlaid map of the structures (white). Image taken by Sentinel-1, preprocessed using SNAP^31^, processed using Python. North is up.

**Figure 14:**
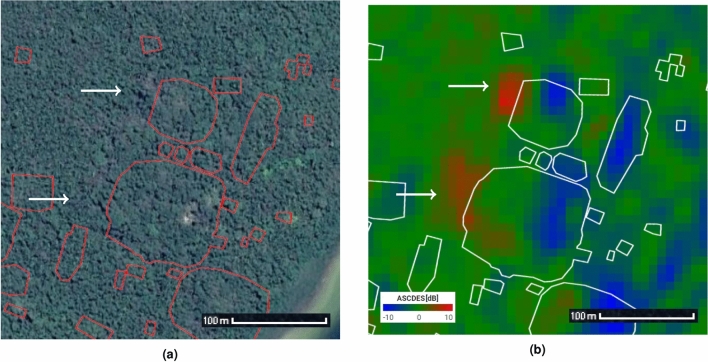
Structures P8-1 (top) and P9-25 (bottom): (**a**) optical image^28^ with overlaid map of the structures (red) (**b**) AscDes ratio with overlaid map of the structures (white). Image taken by Sentinel-1, preprocessed using SNAP^31^, processed using Python. North is up.

This site was interesting for two reasons: because it includes structures that are either visible from space (High Temple, also known as Structure N10-43, Figure 13) or entirely obscured by the forest canopy (Structures P8-1 and P9-25, Figure 14), and because descriptions of the site are available: a map from the 1970s^36^, and buildings dimensions^37^. The three ruins are several tens of meters wide (up to 100m wide for P9-25) and more than 10 meters tall. Numerous smaller structures also cover the site. However, none are as clearly highlighted by our technique as these three. Still, their presence has an impact on the $\Delta \sigma ^\circ$ image.

High Temple (center of Figure 13) is a pyramid cleared of trees. Therefore, we could expect the same sort of signature as for the pyramid of the Sun. However, the latter is massive. High Temple is only 60m wide and 30m tall. With a 10m pixel size, while a triangular signature is still possible, it is also reasonable to expect a less definite shape. As the building is both symmetrical and roughly aligned with the cardinal directions, the red and blue parts of the signature are also symmetrical, with the green line separating them going from north to south.

Structure P8-1 (top of Figure 14) is also a pyramid (according to the map^36^) 75m wide. The signature closely resembles the one from High Temple despite the added canopy. In fact, the ratios on the Western and Eastern sides are more intense here (peak ratios of +5.5/-7.0 dB for High Temple compared to +8.3/-8.8 dB for P8-1). One explanation is that the signature from the edge of the forest around High Temple lessens the temple’s own signature: the west side of the building generates a positive ratio while the trees next to it create a negative ratio (though ground-trunk double-bounces, for example). The resolution and the multi-looking from the GRD preprocessing then tend to merge the two.

Finally, Structure P9-25 (bottom of Figure 14) is described as *an immense platform*^37^, around 100m wide and 18m high. Its signature will, therefore, differ from the pyramids. Its flat top, in particular, appears as a large green area, as expected.

## Discussion

Buildings which are at least a couple of pixels wide (i.e. 20m or larger) and tall enough to backscatter differently than the forest floor are easily visible using our technique. But what about smaller buildings (of the order of a pixel)? We studied the images’ statistics to find a criterion capable of separating “empty” forests from hidden archaeological sites. The first step involved studying the intensity’s histogram. Because the small number of spatial pixels is detrimental to the reliability of statistics, in this section we replaced the temporal averaging with a SAR time series speckle filter developed by the authors^38^ to obtain more data. As the identification technique proposed in this article is not dependent on any particular speckle filter, we expected the produced $\Delta \sigma ^\circ$ images to be qualitatively similar.

120 dates (4 years) were used over Lamanai, and 60 dates (2 years) were used over the sites identified by LaRocque^15^.

**Figure 15:**
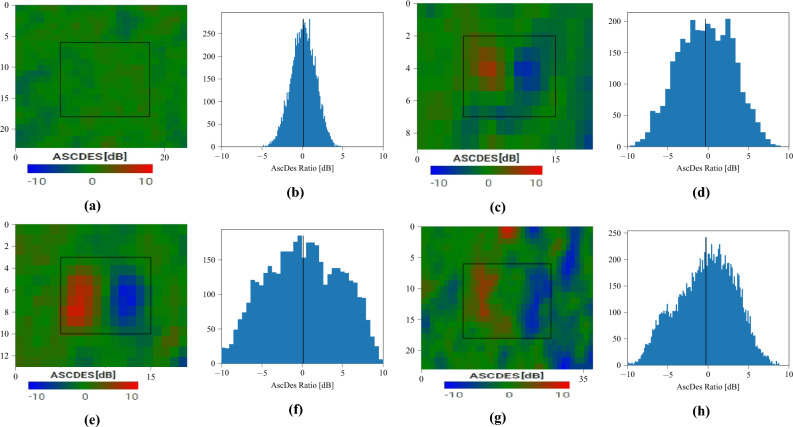
Ratio images (left) and time series histograms (right) over several areas of interest: (**a** & **b**) a forest supposed without hidden structures (**c** & **d**) N10-43 (**e** & **f**) P8-1 (**g** & **h**) P9-25. Black rectangles on the ratio images represent the borders of the AoIs. 120 dates were used in the time series. Some statistics are summarized in Table 3 (left). Every AoI has roughly the same number of pixels. Images taken by Sentinel-1, preprocessed using SNAP^31^, processed using Python. The axes for the images are in pixels. One pixel is 10 m by 10 m. North is up.

The Ascending over Descending ratio (in dB) for an area of forest (Figures 15a and 15b) supposedly devoid of structures follows a centered Gaussian distribution. While a theoretical and perfectly flat canopy would consistently output zero, it is understandable that a practical sensor over a “rough” canopy changing through time would follow such a distribution instead. However, buildings follow a roughly centered non-Gaussian (indicated by a non-zero kurtosis, see Table 3) distribution, whether they’re obscured like P8-1 (Figures 15e and f) and P9-25 (Figures 15g and h) or visible like N10-43 (Figures 15c and 15d). The eastern and western edges produce extreme ratios, which tend to increase the standard deviation for the whole building. The histogram’s centered and symmetrical aspect depends on the structure’s geometry and orientation: a ruin will generate equivalent amounts of positive and negative ratios if correctly oriented and symmetrical, but not if it favors one direction over the other. In any case, the standard deviation and kurtosis seem to be relevant for detection purposes. Their values for each area of interest are summarized in Table 3.

**Table 3:** Statistics of the ascending over descending ratios (in dB) for various areas of interest and over a time series of 120 dates (4 years) for Lamanai and 60 dates (2 years) for LaRocque’s sites. Left to right: the mean, the standard deviation, and the kurtosis. Every AoI has roughly the same number of pixels.

Lamanai	Mean [dB]	StD	Kurtosis
Forest	0.023	1.48	– 0.284
N10-43	– 0.163	3.37	– 0.648
P8-1	0.043	4.53	– 0.795
P9-25	– 0.356	3.52	– 0.643

LaRocque’s	Mean [dB]	StD	Kurtosis
Site A	0.674	2.163	– 0.385
Site B	0.081	2.975	– 0.414
Site C	– 0.087	3.313	– 1.048

The standard deviation highlights not only large individual buildings but also the disturbance caused by a concentration of smaller buildings, i.e., the archaeological site as a whole. By computing this statistic over the whole time series using a (spatial-only) sliding window, it is possible to produce a standard deviation image to better visualize this.

**Figure 16:**
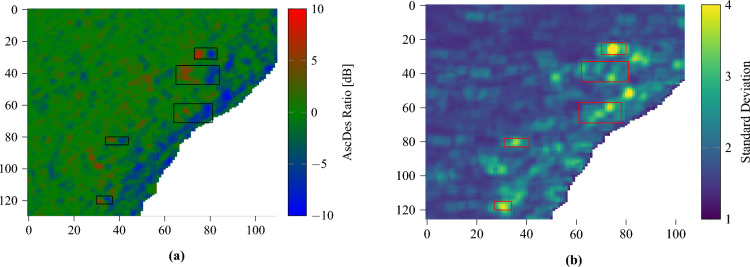
Comparison between the AscDes ratio image (**a**) and the standard deviation of the AscDes ratio over the site of Lamanai (**b**). The window size is the smallest AoI size (i.e., N10-43’s). The lake has been masked out in white. Some known structures are delimited by rectangles. Image taken by Sentinel-1, preprocessed using SNAP^31^, processed using Python. The axes for the images are in pixels. One pixel is 10 m by 10 m. North is up.

Figure 16a is an AscDes ratio image of most of Lamanai. Figure 16b was generated using a 10 by 5 pixels window, which corresponds roughly to the size of N10-43. Higher standard deviation is indeed visible over the area covered by the map^36^. The more significant buildings (some delimited by rectangles) match areas of peak standard deviation. One issue is that the standard deviation is most effective when both the western and eastern sides of buildings are contained within the window. As structures have variable dimensions, computing the standard deviation using an adaptive window size would be beneficial for detection purposes.

Another issue is the difficulty of discriminating between terrain features and buildings. A cliff or steep slope is pretty straightforward, as it is asymmetrical and will, therefore, have a signature containing only either red or blue, depending on its orientation. The only detrimental effect it could cause is to completely obscure the signature from a building on top or near it. On the other hand, small hills, rough terrain or non-uniform canopy (due to non-uniform tree height) would produce positive and negative AscDes ratios, but possibly in a chaotic spatial disposition (because of the various orientations of the slopes, crest lines, etc.). This would make visual interpretation of the AscDes images less reliable. In fact, this kind of terrain would also affect the standard deviation image described earlier.

The only possibility for discriminating terrain features and hidden man-made structures is to count on the higher intensity (and therefore AscDes ratios and standard deviation) caused by double bounces. However, at Sentinel-1 resolution, this scattering mechanism will only have a visible impact on large buildings, buildings that were already easily visually identified.

**Figure 17:**
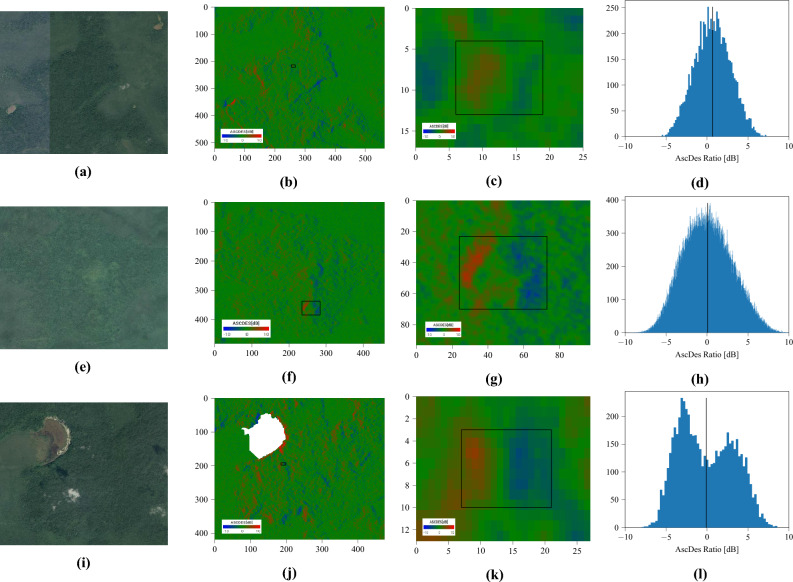
Application over three sites identified by LaRocque^15^. From top to bottom: site A, site B and site C. From left to right: optical image^28^, AscDes ratio image over the whole site, zoom on a potential AoI, and histogram for said AoI. Their borders are represented by black rectangles. A body of water near site C has been masked out in white. 60 dates were used for the time series. Some statistics are summarized in Table 3 (right). Images taken by Sentinel-1, preprocessed using SNAP^31^, processed using Python. The axes for the images are in pixels. One pixel is 10 m by 10 m. North is up.

For example, let us focus on three potential sites identified by LaRocque^15^. Using our technique, Site A (Figure 17, a through d) contains no signature that could positively be a building. The AoI studied for this site (Figure 17c) does follow the red-green-blue order characteristic of a building (although the red and blue areas are darker than expected), its dimensions are similar to some of Lamanai’s structures, and the lower-than-expected ratios (Figure 17d) could be explained by either a worse state of conservation (i.e. fewer double-bounces) or a thicker forest canopy (i.e. less penetration).

The prominent north-south feature in Site B (Figure 17, e through h) could be two platforms linked by a causeway. In fact, the ratios match those from the two platforms under La Danta, a pyramid in the site of El Mirador, Guatemala. However, the dimensions of the potential platforms (Figure 17f, the AoI is almost 500m wide) suggest a naturally occurring terrain feature.

Site C (Figure 17, i through l) also contains a potential detection whose size matches buildings from Lamanai. Its RGB signature (Figure 17k) is more symmetrical in terms of surface, indicating a structure aligned with the cardinal directions. The thin green line indicates a pyramid and not a platform, and the impact of the reduced amount of green pixels is clearly visible on the histogram (Figure 17l, the notch around 0 dB). Like Site A, the ratios are not as intense as in Lamanai, which leads to lower standard deviation (Table 3) than for the three buildings there, even though two of the three structures are smaller than this potential detection.

## Limits

As mentioned in the section “Detection through the forest canopy”, the method proposed in this work relies on an intensity difference between the ascending and descending paths. The difference made the presence of the building stand out from the background (forest, for example). In locations where the signature of the forest is “rough”, i.e. when many small variations of the $\Delta \sigma ^\circ$ value result in a non-uniformly green display, identifying hidden structures with any certainty becomes difficult. If the building’s signature is not obviously different from the background (in terms of ratio value or by appearing as a regular shape), the problem becomes a detection issue where the signature of the forest acts as the noise level.

Another possible source of “false alarms” is small regular terrain features. A small hill covered by trees would have a signature close to the one from a hidden building of similar dimensions. The Bosnian pyramids are a fitting if somewhat extreme example.

One final point we would like to raise is the impact of rotation on the signature. The best source of intensity difference between Ascending and Descending is the double bounce scattering mechanism, as it is the most common source of high intensity backscatter. However, a true “specular” double bounce only happens when the vertical surface of interest is perfectly perpendicular to both the plane of incidence and the ground. This is generally not the case. A rotation of 5^∘^ leads to a drop of at least 20 dB^39^. In particular, Sentinel-1’s trajectory is not perfectly North-South, nor are the buildings studied in this article perfectly aligned with the cardinal directions. However, a less powerful “diffuse” double bounce exists. This mechanism might explain our results. Note however that while the requirements on the rotation angle is lessened, the roughness of the surfaces in play is now of greater importance. Therefore, a perfectly smooth surface hidden in the woods might be harder to detect than our ruins when not perfectly aligned with the SAR line-of-sight.

## Conclusion

In conclusion, the technique enables the identification of potential large structures hidden by a forest canopy. The geolocation of the signatures is acceptable (especially considering the possible shifts between the optical image, SAR image, and the map), and the buildings’ footprints can be retrieved.

The signatures produced could then be segmented using existing algorithms based on edge detection in SAR images^40,41^. Superresolution^42^ might further improve detection performance for structures larger than the resolution. We expect to solve the physical ambiguity on the scattering mechanisms (penetration/no penetration) using the upcoming Sentinel-1C’s Full-Pol data.

### Supplementary Information

Supplementary Information.

## Data Availability

All original Sentinel-1 data are freely available. The datasets generated and analyzed during the current study are available from the corresponding author upon reasonable request.
