# Supplementary material for: Identification of Maya ruins covered by jungle using Sentinel-1
Source: Sci Rep. 2024 Feb 8;14:3293. doi: 10.1038/s41598-024-53068-2 (PMC10853246; doi:10.1038/s41598-024-53068-2)
Supplement: Supplementary file 1 — Supplementary Information. [file 41598_2024_53068_MOESM1_ESM.pdf]

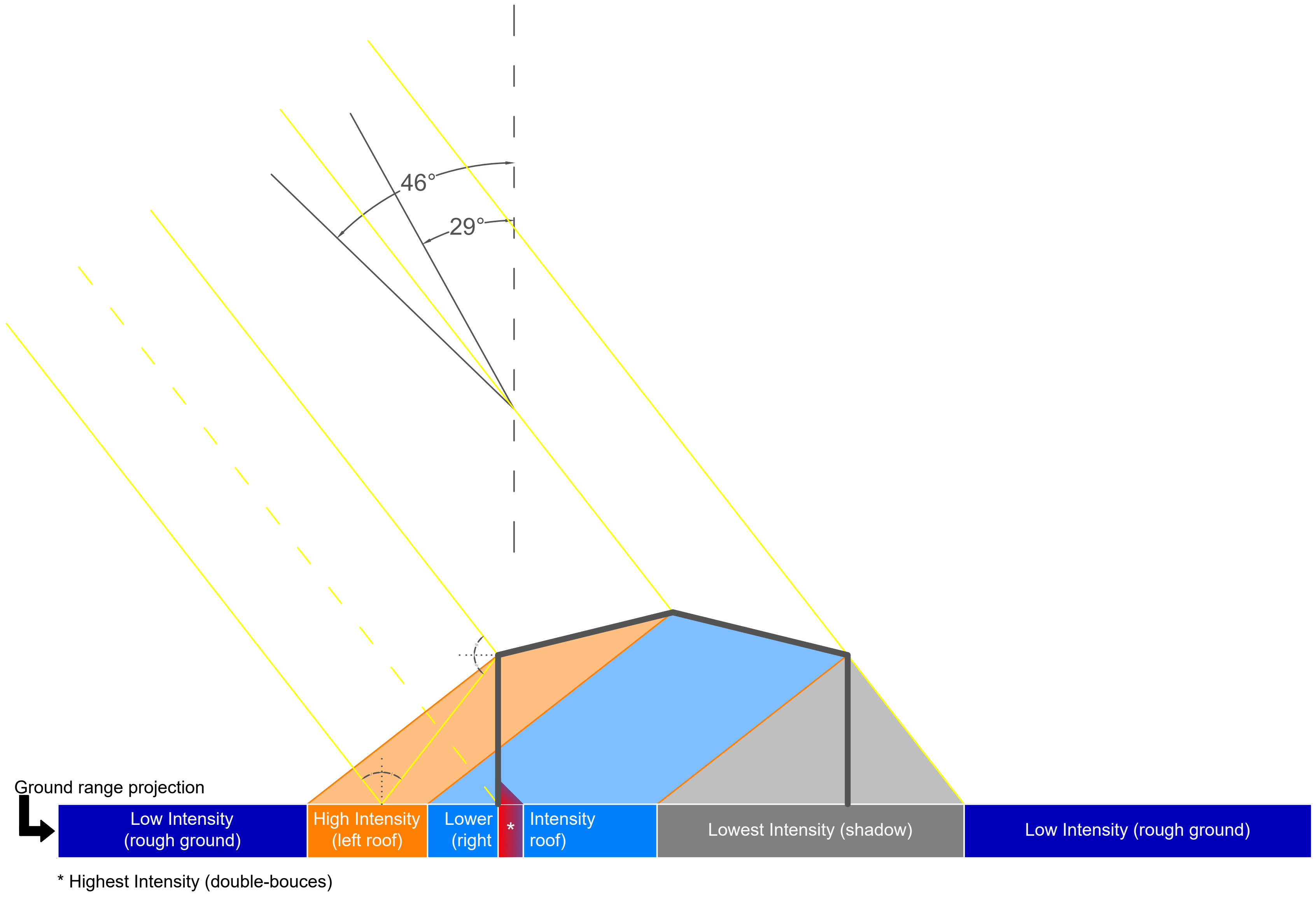
46°
29°
Ground range projection
Low Intensity
(rough ground)
High Intensity
(left roof)
Lower
(right
*
Intensity
roof)
Lowest Intensity (shadow)
Low Intensity (rough ground)
* Highest Intensity (double-bouces)

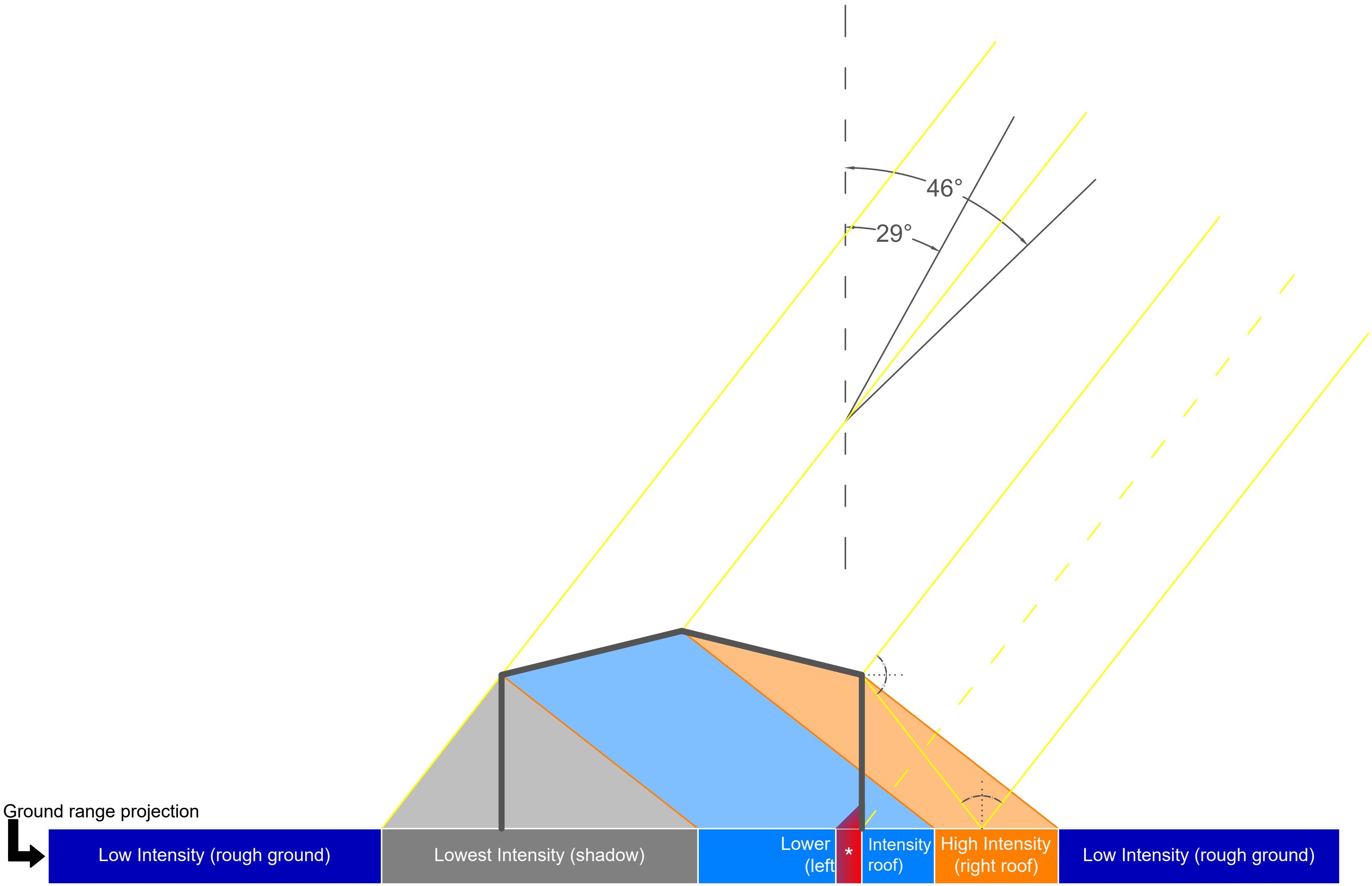

* Highest Intensity (double-bouces)

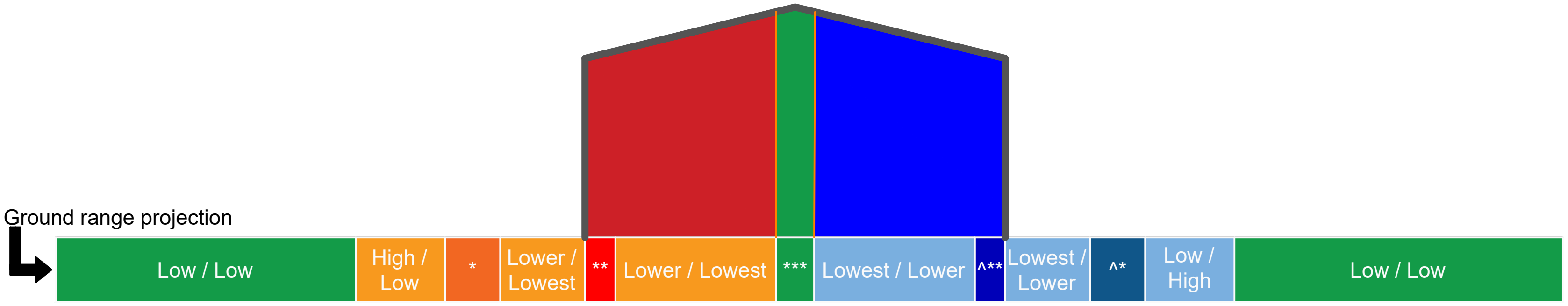

* High / Lowest
** Highest / Lowest
*** Lowest / Lowest
^** Lowest / Highest
^* Lowest / High
